# Investigating the lower ambient temperature limit for pre-cooling to be beneficial for athletic performance

**DOI:** 10.1186/2046-7648-4-S1-A2

**Published:** 2015-09-14

**Authors:** Iris Broekhuijzen, Simon Hodder, Maarten Hupperets, George Havenith

**Affiliations:** 1 Environmental Ergonomics Research Centre, Loughborough University, LE11 3TU, UK

## Introduction

When exercising in the heat, performance is deteriorated. It has been shown that pre-cooling can counteract this deterioration in the heat [1], but it is unclear what the effects of pre-cooling on performance are in temperate environments. Thus, the current study was performed to see if there is any difference in performance with pre-cooling at 24 °C and 27 °C, and thus if there is a threshold in environmental temperature above which pre-cooling becomes beneficial to performance. We hypothesised pre-cooling to enhance performance at both environmental temperatures.

## Methods

Nine healthy males (mean (SD) age 24.2 (7.2) years; VO_2,max _60.6 (6.2) mL.kg^-1^.min^-1^) participated in the study. Six participants performed 4 experimental trials: CON27 (control, 27 °C), COOL27 (pre-cooling, 27 °C), CON24 (control, 24 °C) & COOL24 (pre-cooling, 24 °C). Three participants only performed CON27 & COOL27. Pre-cooling was applied for 30 minutes and during the warm-up before a cycling time trial. Participants were cooled using a cooling vest and sleeves made of a combination of a mesh fabric and pockets filled with hydrophilic silica gel, which was soaked and frozen overnight. 30 minutes of baseline measurements in room temperature (23.3 (0.7) °C) were taken, followed by 39 minutes of pre-cooling in testing climate of which the last 9 minutes participants were warming up. Performance was measured using a time trial equivalent to cycling for one hour at 75 % VO_2,max_. Mean skin temperature (T_skin_) was measured throughout the trial using 8 iButtons [2] and core temperature was measured using a radio pill (T_core_). Body temperature (T_body_) was subsequently calculated using the calculation from Hardy et al. [3]. Thermal sensation (-10 to +10, extremely cold to extremely hot), thermal comfort (0 to 7, comfortable to extremely uncomfortable) and rating of perceived exertion (RPE; [4]) were assessed at 20% intervals of the time trial.

## Results

Results show a significant performance improvement at 27 °C (p = 0.036 (one-tailed)), but no significant differences are seen at 24 °C (p = 0.325 (one-tailed)). This was strengthened by the Hopkins approach [5], which showed a 97% or a very likely chance of an improvement in performance at 27 °C following pre-cooling. Pre-cooling lowered both T_skin _(p < 0.005) and T_body _(p < 0.05), but not T_core_. Sweat rate was significantly lowered following pre-cooling at 27 °C (0.67 (0.11) vs. 0.61 (0.13); p < 0.05), but not at 24 °C (p = 0.075). Furthermore, thermal sensation was lower (i.e. cooler) following pre-cooling (27: 1.6 (1.4) vs. -4.0 (1.41), 24: -0.33 (0.94) vs. -4.33 (1.25); p < 0.05) and thermal discomfort was increased (27: 1.2 (0.4) vs. 2.8 (0.75), 24: 1.0 (0.0) vs. 3.5 (1.2); p < 0.05) following pre-cooling.

## Conclusion

Our results indicate that pre-cooling improves performance in 27 °C, but not in 24 °C and thus that the threshold in environmental temperature for pre-cooling using the tested cooling vest and sleeves to become beneficial for cycling time trial performance appears to be above 24 °C.
